# Unpacking factors influencing antimicrobial use in global aquaculture and their implication for management: a review from a systems perspective

**DOI:** 10.1007/s11625-017-0511-8

**Published:** 2017-11-18

**Authors:** Patrik J. G. Henriksson, Andreu Rico, Max Troell, Dane H. Klinger, Alejandro H. Buschmann, Sonja Saksida, Mohan V. Chadag, Wenbo Zhang

**Affiliations:** 1 Stockholm Resilience Centre, Stockholm University, Kräftriket 2B, 10691 Stockholm, Sweden; 2 WorldFish, Jalan Batu Maung, Batu Maung, 11960 Bayan Lepas, Penang Malaysia; 3 IMDEA Water Institute, Science and Technology Campus of the University of Alcalá, Avenida Punto Com 2, P.O. Box 28805, Alcalá de Henares, Madrid Spain; 4 The Beijer Institute of Ecological Economics, The Royal Swedish Academy of Sciences, Box 50005, 104 05 Stockholm, Sweden; 5 Department of Ecology and Evolutionary Biology, Princeton University, Princeton, NJ 08544 USA; 6 Centro i-mar and CeBiB, Universidad de Los Lagos, Puerto Montt, Chile; 7 Aquaculture Management Division, Fisheries and Oceans Canada, Ottawa, Canada; 8 College of Fisheries and Life Science, Shanghai Ocean University, Shanghai, 201306 China

**Keywords:** Antimicrobials, Antibiotics, Aquaculture, Seafood, Resistance, Fish

## Abstract

Global seafood provides almost 20% of all animal protein in diets, and aquaculture is, despite weakening trends, the fastest growing food sector worldwide. Recent increases in production have largely been achieved through intensification of existing farming systems, resulting in higher risks of disease outbreaks. This has led to increased use of antimicrobials (AMs) and consequent antimicrobial resistance (AMR) in many farming sectors, which may compromise the treatment of bacterial infections in the aquaculture species itself and increase the risks of AMR in humans through zoonotic diseases or through the transfer of AMR genes to human bacteria. Multiple stakeholders have, as a result, criticized the aquaculture industry, resulting in consequent regulations in some countries. AM use in aquaculture differs from that in livestock farming due to aquaculture’s greater diversity of species and farming systems, alternative means of AM application, and less consolidated farming practices in many regions. This, together with less research on AM use in aquaculture in general, suggests that large data gaps persist with regards to its overall use, breakdowns by species and system, and how AMs become distributed in, and impact on, the overall social-ecological systems in which they are embedded. This paper identifies the main factors (and challenges) behind application rates, which enables discussion of mitigation pathways. From a set of identified key mechanisms for AM usage, six proximate factors are identified: vulnerability to bacterial disease, AM access, disease diagnostic capacity, AMR, target markets and food safety regulations, and certification. Building upon these can enable local governments to reduce AM use through farmer training, spatial planning, assistance with disease identification, and stricter regulations. National governments and international organizations could, in turn, assist with disease-free juveniles and vaccines, enforce rigid monitoring of the quantity and quality of AMs used by farmers and the AM residues in the farmed species and in the environment, and promote measures to reduce potential human health risks associated with AMR.

**Electronic supplementary material:**

The online version of this article (10.1007/s11625-017-0511-8) contains supplementary material, which is available to authorized users.

## Introduction

Antimicrobials (AMs) are defined as pharmaceuticals that kill or inhibit the growth of microorganisms and include antibiotics (AB), antivirals, antifungals, and antiprotozoal substances. While their use is at the foundation of modern medicine, escalating use has increased the risk of antimicrobial resistance (AMR) in pathogenic and non-pathogenic bacteria, leading to a reduced treatment efficacy of AMs for diseases such as pneumonia, tuberculosis, and gastrointestinal infections (Fair and Tor 2014). These diseases are thought to have been responsible for 30% of the human deaths in the US in the pre-AM era (Fair and Tor 2014). AMR also threatens modern surgical procedures that rely upon effective AMs for post-operative care (Teillant et al. 2015). Consequently, the spread of AMR bacteria has been classified by the World Health Organization (WHO) as one of the major threats for the human population of the twenty-first century (WHO.int; accessed 21-Jan-2017).

Over the past decade, AMs have been increasingly used in animal production to prevent and treat disease, and also as growth promoters (Van Boeckel et al. 2015). Consequently, AM residues have been detected in terrestrial, freshwater, and marine environments near agriculture and aquaculture facilities (Husevåg et al. 1991; Samuelsen et al. 1994; Smith et al. 1994; Capone et al. 1996; Chelossi et al. 2003; Boxall et al. 2004; Baquero et al. 2008; Kümmerer 2009; Rico et al. 2014b; Hatosy and Martiny 2015; Nakayama et al. 2017). AMs and their breakdown products are most often released into the environment via discharge of human sewage, livestock and aquaculture run-off, or through the spread of manure over agricultural lands (Sarmah et al. 2006; Marshall and Levy 2011; Andrieu et al. 2015; Robinson et al. 2016). In aquaculture systems, AMs are commonly applied with feed or directly to the water and may later be released into the environment through run-off water or sedimentation of feces and uneaten feed particles, which are then consumed by nearby fish or invertebrates (Capone et al. 1996; Fortt et al. 2007; Buschmann et al. 2012; Rico et al. 2013; Rico and Van den Brink 2014; Andrieu et al. 2015; Muziasari et al. 2017). Several studies have shown that AM releases from aquaculture facilities can contribute to increased risk of AMR development in environmental compartments (Tendencia and De La Peña 2001; Le et al. 2005; Sun et al. 2016; Nakayama et al. 2017; Rico et al. 2017) and how the aquatic environment can become a source of AMR bacteria and AMR genes that can pose risks to humans (Rhodes et al. 2000; Poirel et al. 2012; Aedo et al. 2014; García-Aljaro et al. 2014; Xu et al. 2017).

A joint study by the European Centre for Disease Prevention and Control (ECDC), European Food Safety Authority (EFSA), and the European Medicines Agency (EMA) (ECDC/EFSA/EMA 2015) on AM agents and occurrence of AMR identified a positive correlation between the overall use of AMs in animal husbandry and the occurrence of AMR genes in human pathogens. The same investigation also revealed that the use of AMs in animal husbandry is much higher than in human medicine, even for some AMs that are deemed critically important in human medicine (i.e. cephalosporins and quinolones). Moreover, there is a potential bridging between aquatic and human pathogen resistomes that leads to emergence of new AMR bacteria and the dissemination of their AMR genes into animal and human populations (Cabello et al. 2016). For example, a recent study reported significantly higher frequencies of AMR genes in urinary *Escherichia coli* isolates from Chileans living in aquaculture regions compared to isolates from non-aquaculture localities, suggesting that AM use in the Chilean salmon industry may be contributing to increased risks of AMR genes in humans (Tomova et al. 2015). AM use in animal husbandry, therefore, has repercussions for humans, while many AMs also are directly toxic to non-pathogenic bacteria and primary producers, possibly affecting ecosystem functions and biochemical processes mediated by microorganisms (Christensen et al. 2006; Rico et al. 2014a; Guo et al. 2015).

**Fig. 1:**
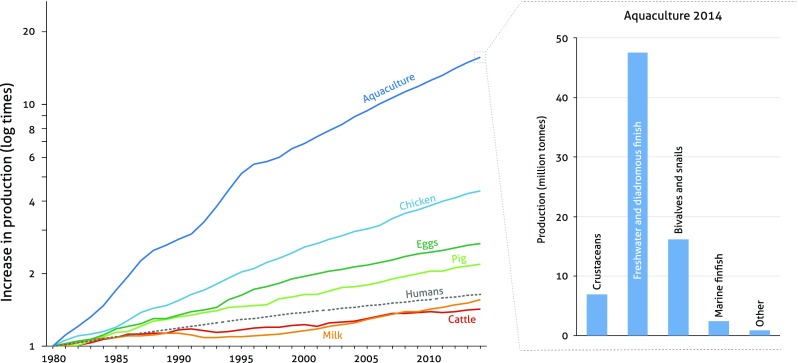
Proportionate growth of the human population, aquaculture, poultry, pigs, and cattle between 1980 and 2013, and the present composition of the aquaculture sector *Source* (FAO 2016a; The World Bank 2017; FAO 2016b)

A few studies have tried to quantify global AM use (e.g. Done et al. 2015; Van Boeckel et al. 2015), but all face limitations related to their breakdown of consumption by animal species and production systems, the reason for this being the majority of AMs used in different food-production commodities remain mostly unmonitored (Done et al. 2015). Building upon the limited data available on specific usage patterns, a recent study by Van Boeckel et al. (2015) anticipated that global consumption of AMs in livestock production could increase by as much as 67% between 2010 and 2030. This study, however, did not quantify AMs used in aquaculture, the fastest growing animal food production sector at present (Fig. 1) (Troell et al. 2014; Henriksson et al. 2015).

Aquaculture represents slightly less than 10% of total farmed animal production by volume, but production is expected to double over the coming two decades (The World Bank 2013; FAO 2016a, b). Asia dominates global aquaculture production, with China providing more than 60% of global fish supply (finfishes, crustaceans, and bivalves) (FAO 2016a). Although several studies have reported on the use of AMs in specific aquaculture systems and regions (Burridge et al. 2010; Rico et al. 2012; Cabello et al. 2013; Rico et al. 2013; Done et al. 2015; Ali et al. 2016), no comprehensive overview of the sector’s global use of AMs and the underlying drivers exists. This implies that risks and benefits of antibiotic usage cannot be adequately assessed (Done et al. 2015).

Evaluations of national or global AM use in aquaculture is complicated by numerous factors, including the diversity of species and culture systems, the unconsolidated nature of production in many regions (i.e. many independent small producers in an area), and the often unregulated use of AMs that are labeled and registered for use in livestock and aquaculture production. Most available AM use datasets only describe high-value aquatic species in high-income countries and are often based on countrywide sales (e.g. salmon in Norway) (Burridge et al. 2010; Grave and Brun 2016). In contrast, quantitative information from Asian countries is often based on extrapolations from isolated farmer surveys (e.g. Rico et al. 2013; Ali et al. 2016). This has contributed to a rather confusing, and sometimes biased, representation of overall AM use by sectors, as application rates vary greatly across different countries, regions, years, application methods, and aquaculture species (e.g. Rico et al. 2013; Henriksson et al. 2015). For example, in 2013 AM use in Norwegian salmon farming was 1.3 g tonne^−1^ of harvested product, whereas in British Columbia (Canada) it was 43.7 g tonne^−1^, and in Chile it was 701 g tonne^−1^ (Bridson 2014). In another example, Le and Munekage (2004) quantified the number of AB types used among Vietnamese shrimp farmers and reported the use of 11 different ABs in 2004, while a similar survey performed on 34 shrimp farmers between 2011 and 2012 found that only oxytetracycline was used, and only by a single farmer (Rico et al. 2013).

The main objective of the present report was to identify and describe drivers that could explain the interspecies, intersystem, and/or trans-regional differences in AM use in global aquaculture and identify possible mechanisms for reducing AM use in social-ecological systems. Moreover, a methodological approach for the identification of future AM usage scenarios and excessive or unregulated AM ‘hotspots’ is also proposed, based on governance indices. The overall aim is to help identify areas in which immediate action is required for the establishment of AM use evaluation and to identify environmental and human health regulation schemes, while providing recommendations for diminishing AM use and risks in the future. We do not quantify AM use in aquaculture or systematically review our molecular and evolutionary understanding of AM ecology.

## Main drivers behind AM use in aquaculture

Below we identify the primary drivers behind suboptimal AM use, from the animal level (Sect. “Species vulnerability”), the farm level (Sect. “Production practices and technology”), the regional level (Sect. “Regional vulnerability”), and the institutional level (Sect. “Institutional vulnerability”).

### Species vulnerability

Aquaculture is an evolving agri-business with over 600 different species under cultivation, drawn from the full spectrum of trophic levels and cultured using a wide range of technologies and inputs (FAO 2016a). New species are also being tested for their potential in intensive farming systems, unlike terrestrial animal farming, which largely utilizes less than a dozen culture animal species (albeit with different breeds) (Troell et al. 2014; FAO 2016b). The recent trend in aquaculture development has, however, been towards intensification (concentration) of a smaller set of aquatic species. At present, about 44 ectothermic species make up 90% of total global aquaculture production (compared to only five for livestock) (Troell et al. 2014; FAO 2016a, b), with most originating from tropical and sub-tropical regions that are generally more prone to bacterial disease outbreaks (Leung and Bates 2013). Immune responses and capacities, however, differ greatly among different types of farmed aquatic organisms, with only vertebrate species having an adaptive immune system that can produce antibodies to combat bacterial infections (Du Pasquier 2001). For bivalves and snails, AM use is generally restricted to hatcheries out of practical reasons, as these are filter-feeding or grazing organisms farmed in open environments (Paillard et al. 2004). AMR genes have, however, been found in adult bivalves farmed in proximity to other finfish aquacultures (Collado et al. 2014).

While the transfer of existing technologies and husbandry practices (e.g. rearing densities, feeding, development of vaccines, and feeds) from more established species to new species can be beneficial, the large diversity of aquaculture species presents challenges to understanding disease vulnerability, immune responses, and development of vaccines (Brudeseth et al. 2013). For example, over the past 20 years in Pacific Canada and the US there has been a shift from primarily farming two indigenous species of *Salmonidae*, Chinook and Coho Pacific salmon (*Oncorhynchus* spp.), towards an almost total dominance of the introduced Atlantic salmon (*Salmo salar*). The main driver behind this shift has been disease management, specifically control of Bacterial Kidney Disease that affects Pacific salmon more severely than Atlantic salmon (Morrison and Saksida 2013). The shift from Pacific to Atlantic salmon consequently resulted in a tenfold decrease in AB use (oxytetracycline) in salmon farming, which was also helped by available vaccines and better farming practices (Morrison and Saksida 2013).

Another example from the late twentieth century is the shift from traditional coastal brackish-water shrimp systems to semi-intensive and intensive shrimp farming systems in Asia. This intensification resulted in increased disease outbreaks (including *Vibrio* spp. bacteria) throughout Asia, with consequent additional use of AMs (Kautsky et al. 2000). Disease epizootics in Asia (e.g. White Spot Disease and Yellowhead disease) have fueled a dramatic increase in the use of chemicals and AMs in intensified Penaeid shrimp grow-out systems, while, in the past, they had primarily been used in hatcheries. Inaccurate and incomplete disease diagnostics by farmers (e.g. AM treatment of diseases caused by viral pathogens), lack of stringent national policies on the use of AMs for fish and shellfish, and easy accessibility to AMs all contributed to a massive surge in the use of AMs by the aquaculture industry. Since shrimp is a commodity primarily destined for export, importing countries strengthened residue-testing programs for AMs, resulting in rejections of shipments and even an EU ban on Thai shrimp imports after the detection of chloramphenicol and nitrofurans residues in 2002 (Lebel et al. 2010; Rico et al. 2013). This led to a rapid transition in the Thai shrimp sector from farming the indigenous Asian tiger shrimp (*Penaeus monodon*) to farming whiteleg shrimp (*Litopenaeus vannamei*), for which specific-pathogen-free (SPF) juveniles were available (Lebel et al. 2010). Together with improved farming practices, this reduced the reported AB use frequency among Thai farmers from 78% of shrimp farmers in 2000 to only 3% in 2011–2012 (Holmström et al. 2003; Rico et al. 2013). While there is an inherent risk of underreporting in farmer self-reporting studies, especially after farmers are made aware about controversies related to AMs use, the latter of these studies was correlated by a supporting survey of farm supply shops (Rico et al. 2013). However, since these improvements, new diseases have recently emerged, such as Acute Hepatopancreatic Necrosis Disease (AHPND) and Enterocytozoon hepatopenaei (EHP), in both Asian tiger and whiteleg shrimps, which may incentivize increases in future use of AMs in shrimp farming (Li et al. 2016).

Thus, the fact that susceptibility to disease and symptoms vary across species and strains points to the need to find better ways to manage and administer AMs. Properly diagnosing disease and understanding how different organisms’ immune systems respond to pathogens is, therefore, key to reducing both mortalities and AM use. Closing the biological lifecycle of a farmed species is also essential for developing less susceptible strains and SPF juveniles. The latter could, together with stricter control of regional and national transboundary movement of live animals for aquaculture (e.g. broodstock and seed), contribute to reduced use of AMs. This could, for example, have prevented the global spread of the recently discovered tilapia lake virus (TiLV), which has already been detected in Israel, Ecuador, and Egypt (Bacharach et al. 2016; Fathi et al. 2017), where farmers, unaware of it being a viral disease, easily will turn to AMs.

### Production practices and technology

Freshwater pond aquaculture produces the majority of finfish today (Fig. 1) (FAO 2016a), but reservoirs, tanks, lakes, rivers, and canals are also used for freshwater farming. Cage culture is the most common form of marine finfish aquaculture, while crustaceans are predominantly farmed in brackish and freshwater ponds (Hall et al. 2011; MOA 2016). While cages and many ponds benefit from natural water exchange for oxygen provision and waste removal, they are simultaneously more exposed to disease causing agents that occur in the water. In response, new technologies have been developed in an effort to better control the farming environment, including closed recirculating aquaculture systems (RAS) that reduce wastes and disease vectors (Martins et al. 2010).

Independent of farming system, farmers may choose to use AMs either for treatment or as a prophylactic, a choice dependent on many factors including a farmer’s own knowledge. However, unlike livestock farming, prophylactic use in aquaculture is generally to prevent mass mortalities rather than to promote growth (Cabello et al. 2013). Concerns of increasing prophylactic use of AMs in shrimp, salmon, and other farming sectors have been reported (Cabello et al. 2013; Watts et al. 2017). In contrast, Rico et al. (2013) reported that only 5% of pangasius (*Pangasius* spp.) farmers in Vietnam used ABs as a prophylactic measure, and the Vietnamese Ministry of Agriculture and Rural Development recently launched even stricter guidelines for the coming years (fao.org/antimicrobial-resistance/news-and-events/news/news-details/en/c/1027602/, accessed 18-Aug-2017). This may indicate differences in practices among species, farming systems, and countries. Vietnamese pangasius farming in Vietnam may, for example, represent a special case due to extensive certification initiatives and constant progress towards improved disease diagnosis and management, particularly in large-scale enterprises (Nhu et al. 2016).

Other factors that can influence emergence and spread of diseases include water quality parameters, such as dissolved oxygen, turbidity, temperature, nutrition, age, source of juveniles, and the presence of disease vectors (where zooplankton, birds, crabs, and snails are the most common) (Piasecki et al. 2004; Clausen et al. 2015). Improved farming practices and management can, therefore, help reduce exposure and susceptibility to disease and subsequent reliance on AMs (Defoirdt et al. 2011; Romero et al. 2012). Additionally, spatial planning of farm activities at a more regional scale has the potential to better mitigate aquatic spread of diseases across farms, involving measures such as water control and reuse, and synchronized farming activities (Kautsky et al. 2000; Bondad-Reantaso et al. 2012; Guerry et al. 2012). In conclusion, many disease outbreaks could be avoided by improved farming practices and sanitary conditions.

### Regional vulnerability

**Fig. 2:**
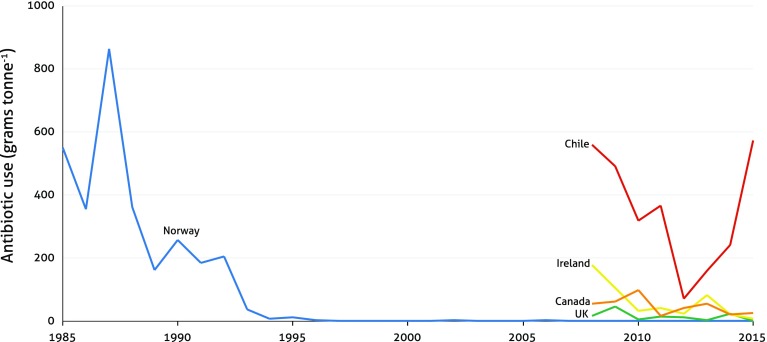
Antibiotic use in Atlantic salmon farming in the five top producing countries Data from Marine Harvest (2015) and R. Gudding, Norwegian Veterinary Institute, Pers. Comm. (2013)

Different geographical regions can host different bacterial pathogens at different densities, which in turn influences the use of AMs. For example, Atlantic salmon farms in Chile are subject to the increased presence of *Piscirickettsia salmonis*, a bacterial disease that causes Salmon Rickettsial Syndrome (SRS), which can lead to massive die-offs if left untreated (Rozas and Enríquez 2014). SRS outbreaks are less common in Atlantic salmon farms in northern Europe, most likely due to environmental conditions that are less conducive to *P. salmonis* or its possible vectors, better quality of juveniles (smolts), or better overall management (Rozas and Enríquez 2014). AM use in salmon farms in Chile, therefore, exceeds usage in salmon farms elsewhere (Fig. 2) (Rico et al. 2013; Bridson 2014; Marine Harvest 2015). It was also recently demonstrated that SRS in Chile has developed an up to 200-fold increase in AMR, which has resulted in escalating AM use (Henríquez et al. 2015).

### Institutional vulnerability

Beyond the physiological drivers for AM use, institutional shortcomings may be directly linked to excessive AM use. Institutional drivers range from access to retailers selling AMs, access to veterinarians, legislation, and enforcement of legislation, to custom controls in importing countries and certification schemes.

While many countries have limits for acceptable AM residues, some countries only employ enforcement programs on export products, meaning products meant for domestic consumption are less likely to be tested (Boison and Turnipseed 2015). China, for example, maintains two almost separate production chains, one for the domestic market and one CIQ-registered (China Inspection and Quarantine) aimed for export (see Tables S1 and S2 in the Supporting Material for a list of banned therapeutants and allowed ABs in China). The CIQ system started operating in 2002, both for farmed and captured products. While the list of illegal chemicals apply to all seafood, not just exported seafood, the “Measures for the Supervision and Administration of the Inspection and Quarantine of Export-Oriented Aquatic Animals” (AQSIQ 2007) originally indicated that the government will only regularly check for AM residues in those products aimed for export (following the regulations of the importing country). However, recent announcements for food safety inspection published by the China Food and Drug Administration (sda.gov.cn/WS01/CL1664/, accessed 29-Aug-2017) state that inspections of seafood sold on the domestic market commenced in 2013. In many other lower income countries, monitoring and enforcement of regulations related to seafood have also been hampered by the distribution of jurisdiction and responsibilities among numerous government departments (Mo et al. 2017).

**Fig. 3:**
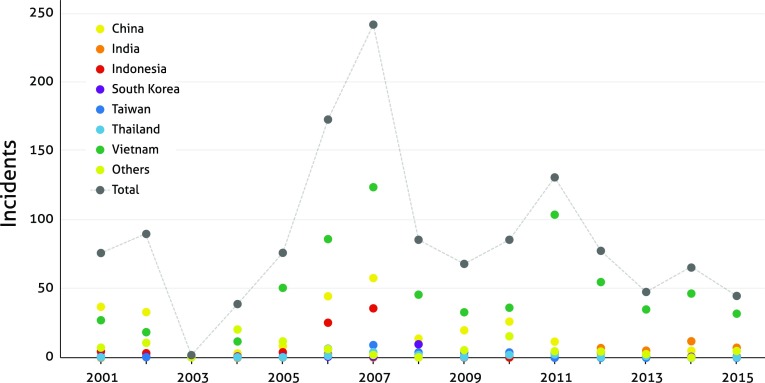
The number of reported incidents in EU, US, and Japanese customs involving antimicrobial residues. The data are not reported consistently, explaining some of the discrepancies and why no cases were reported in 2003 *Sources* EU’s Rapid Alert System for Food and Feed, US’s Food and Drug Administration, and Japan’s Ministry of Health, Labour and Welfare

Analysis of the number of incidents involving AM residues in imported seafood products, as reported by the EU’s Rapid Alert System for Food and Feed, the US’s Food and Drug Administration, and Japan’s Ministry of Health, Labour, and Welfare, provides a crude insight into how countries and sectors differ with regards to AMs use (Fig. 3 and Table S4). These data are not reported annually and sometimes only per consignment, resulting in inconsistency of reported incidents (e.g. no data were reported in 2003). Other concerns related to these data include their limitation to export-oriented products, re-exports, inconsistent sampling with regard to years and countries, and constantly evolving measurement instruments. Despite these shortcomings, there are certain trends in AM use that stand out and that coincide with particular events. For example, Thailand, one of the most innovative and leading aquaculture countries in Asia (Lebel et al. 2010), has only a few reported cases of AM residues since 2003. Indonesia, on the other hand, had an upsurge of incidents between 2005 and 2009, possibly a consequence of a Koi herpes virus outbreak around the same time that was misdiagnosed as a bacterial pathogen, resulting in increased AM use (Sunarto and Cameron 2005). Incidents in India have also increased in recent years (primarily due to continued use of banned furazolidone and nitrofurazone), reflecting a surge in whiteleg shrimp farming and exports (FAO 2016a). Vietnam had the highest number of incidents in the last decade, with traces of 18 different AMs reported in shrimp and pangasius products, supporting the extensive list of AM compounds reported to be used in that country by Rico et al. (2013), Phu et al. (2016), and Thi Kim Chi et al. (2017).

## Mechanisms to control AM use

At present, uninhibited and uninformed use of AMs is adding to the problem of AMR, which in turn fuels AM use. The challenge is to break this vicious circle. Below we discuss mechanisms that could be used to reduce AM use, based on the underlying factors identified in the previous section.

### Biosecurity

Biosecurity refers to any step that would prevent entry of pathogens into farms or hatcheries, thereby reducing the risk of disease outbreaks and consequent AM use (Bondad-Reantaso et al. 2012). On pond-based grow-out farms, simple biosecurity measures would include deterrents to keep disease vectors out (such as bird nets and scares, or barriers for crabs), drying of sediments, liming of ponds, and organic waste removal before re-stocking (Yanong 2013). Excessive organic waste build-up serves as a reservoir for bacteria and other microorganisms, while organic loading increases the biological oxygen demand (Yanong 2013). Equipment, such as seine nets, paddle wheels, and vehicles, can also harbor and carry infectious disease between ponds and farms, a risk that can be reduced by adequate cleaning, disinfection, and/or drying between uses (Yanong 2013). More elaborate physical biosecurity measures also involve mechanical aeration and water treatment (e.g. by use of pre- and probiotics) to reduce water exchange rates from local waterways, implying that the risk of transferring vectors and associated pathogens will be minimized. An extreme example of an increasingly used biosecurity system is RAS, where disease vectors can be almost completely excluded through water treatment and re-use. RAS systems, however, still carry the risk of disease exposure through the introduction of new animals and feeds (Martins et al. 2010).

Implementing biosecurity programs at hatcheries can help reduce the incidence of disease. Screening post-larvae coming out of SPF hatcheries for viral pathogens, using rapid testing and screening tools (e.g. PCR), is a preferred option that can ensure absence of pathogens. For example, highly biosecure hatcheries produce SPF Asian tiger shrimp post-larvae that can reduce farm exposure to disease (Bondad-Reantaso et al. 2012).

At a regional level, coordinated planning may further help reduce overall disease prevalence if farms are sited sufficiently far apart to reduce disease transfer (Guerry et al. 2012). Regional management initiatives, including rapid response mechanisms, stronger regulations (e.g. maximum production and biomass loads, or organic waste management protocols), and comprehensive strategies for responsible introduction of live aquatic animals (e.g. ICES Code of Practice on the Introductions and Transfers of Marine Organisms) may also help reduce the risk of disease outbreaks and limit the spread of pathogens.

### Aquaculture extension programs

Extension programs are aimed at helping farmers improve farming practices and are generally issued by governments and NGOs (Garrett et al. 1997). Many aquaculture extension programs have been launched to date, but their reach is limited in many lower income regions. In the meantime, much of the overuse of AMs is directly related to uninformed farmer decisions, including incorrect diagnoses, excessive dosage (sometimes due to illiteracy, miscalculations, or having experienced treatment failure at recommended dosages), use of poor quality feeds or drug products, overfeeding, insufficient infrastructure, and poor water management (Garrett et al. 1997). The promotion of probiotics and greenwater techniques, which boost the growth of health-promoting bacteria in cultured animals, has also proven to be an efficient mean of preventing AM use by reducing pathogenic bacteria and animals’ susceptibility to disease (Balcázar et al. 2006; Natrah et al. 2014; Bentzon-Tilia et al. 2016). It has been argued that AMs have negative long-term effects on pond microbial systems in general, by destroying healthy bacterial communities (Lavilla-Pitogo et al. 1998; De Schryver et al. 2014). De Schryver et al. (2014), for example, suggests that AM use (especially prophylactic use) may increase the prevalence of outbreaks of *Vibrio* spp. in shrimp cultures by destroying mature microbial systems of slow-growing bacteria in the pond water, thereby benefitting faster growing bacteria (incl. *Vibrio* spp.) that often are pathogenic.

Water temperature is also directly linked to the growth of pathogens, and, as most fish are ectotherms, they use temperature gradients to induce behavioral fever (Cerqueira et al. 2016). This behavioral response is, however, hampered in ponds and cages that restrict the fish’s movement. Thus, providing temperature gradients in the farm medium by helping farmers construct ponds with alternating depths and/or partial shading could help limit the severity of disease outbreaks (MacKenzie, pers com.). Climate change will further influence temperature and consequently the type, spread, and frequency of disease (Burge et al. 2014). This stresses the importance of improving the resilience of the aquaculture industry by maintaining suitable genetic, species, and farming diversity to match variability in the environmental conditions of the production area (Troell et al. 2014; Klinger et al. 2017).

Improved farm management can be achieved through extension programs that improve information flows. Extension agencies can help communicate the risks of misuse and overuse and demonstrate the efficacy of judicious use, best use practices, alternative treatment options, and disease avoidance techniques (Hernández Serrano 2005), all of which can limit AM use. While extension agencies are common throughout most higher income countries, similar programs are often absent or underfunded in lower income countries where most aquaculture currently takes place (Aker 2011). Top-down extension efforts can also be unsuccessful at engaging farmers and other supply chain actors if they do not allow for bidirectional communication and grassroots participation, further complicating extension in lower income countries (Umesh et al. 2010).

### Improved farm support for diagnostics and treatment

The capacity to accurately diagnose disease is essential to effective treatment. Challenges associated with new species, established species in new environments, and new diseases may lead to delayed diagnosis and treatment or treatment without a proper diagnosis. Most high and many upper middle income countries have veterinarians, technicians, diagnostic labs, and research facilities available to provide fish health advice, but in lower middle income countries the lack of disease diagnostic capacity, including fish health experts, hinders rapid and proper diagnosis, and often leads to inappropriate use of AMs. For new or emerging diseases, however, diagnostics will remain obstructed until confirmatory diagnostic methods become available. These confirmatory diagnostics should preferably enable farmers to test their animals and determine a diagnosis, as has been achieved by, e.g. IE WSSV and YHV strip test (Wangman et al. 2016).

Increased frequencies of AMR may decrease the effectiveness of AM treatments, and, if farmers respond to this decrease in efficacy by using greater amounts of AMs, the resistance itself becomes a proximate driver of increased usage. The lack of AM alternatives thus easily results in AM abuse. Rotating the active ingredient has been proposed for reducing the likelihood of such situations, by avoiding selection and co-selection of AMR genes (Niederman 1997; Miranda et al. 2013). Clinical studies have, however, shown limited success for this strategy, as AMR genes can persist for long periods after the removal of the relevant selection pressure, which in this case would be any specific active ingredient (Taylor et al. 2011; Lee et al. 2013). Ecological theory instead suggests that mixing compounds actually yields better results than cycling (Bergstrom et al. 2004; Levin and Bonten 2004). Although AM mixing has shown great potential, random mixing without proper disease diagnosis and previous toxicological tests might result in inappropriate usage of AMs, thus suggesting that more clinical studies are needed (Lee et al. 2013). Nevertheless, excessive use or abuse of AMs will lead to an alteration of the resistome in target and non-target bacteria in environments surrounding farms and potentially spread to downstream farms. The problem of AM use and AMR, therefore, needs to be tackled internationally by adopting one common approach, to avoid the spread of AMR genes, including a coordinated ‘One Health’ approach with the livestock sector and human medicine practitioners (AVMA 2008; onehealthinitiative.com, accessed 29-Aug-2017).

### Limiting AM access

Changes in access to AMs can be due to regulations that either permit or do not explicitly disapprove the use of some substances. AMs may also be inaccessible to farmers due to costs or lack of access to pharmaceutical markets or distribution networks. The most efficient ways of regulating access to AMs largely varies among countries.

Most upper middle and high income countries have lists of explicitly approved AMs. For example, in Canada there are four AM products registered for aquaculture, containing: oxytetracycline, florfenicol, trimethoprim/sulfadiazine, and ormetoprim/sulfadimethoxine (DFO 2017). These products are administered through medicated feeds and require veterinary prescriptions. Other AMs may be obtained through an ‘Emergency Drug Release’ provided by veterinarians in special cases (Health Canada 2017), such as erythromycin use in broodstock. The importance of oxolinic acid and flumequine (quinolone AMs) in human medicine has led to a prohibition of their use for treating salmon in Canada and Scotland (Burridge et al. 2010). In the US, the US Food and Drug Administration regulates AM use in aquaculture with specific applications for specific species and only three approved ABs (florfenicol,oxytetracycline, and sulfamethoxine/ormethoprim) (USFDA 2017).

Other countries, from our experience, take an alternative approach and explicitly ban AM substance groups that are known or suspected to cause carcinogenic or mutagenic effects in consumers (i.e., nitrofurans, nitroimidazoles, malachite green, and chloramphenicol and its derivatives), while use of non-banned AMs is tacitly allowed. Liu et al. (2017), for example, describe a Chinese ban on erythromycin in 2002, but also report continued use in 2012 based on literature sources. In the same review, Liu et al. (2017) also document 20 different ABs being used in Chinese aquaculture, while only 13 ABs were authorized. Thailand, on the other hand, only approves the use of five AM substances (enrofloxacin, oxytetracycline, sulfamethoxine/ormethoprim, and amoxicillin) (Baoprasertkul and Somsiri 2012), while Vietnam approves 27 different active ingredients, including substances used in human medicine (VMARD 2012). Rico et al. (2013) also reported 17 different AB compounds being applied in pangasius aquaculture, belonging to ten different AB classes, some which also are of critical relevance for human medicine (e.g. Kanamycin) (WHO 2011). Three years later, Ali et al. (2016) identified seven different ABs being used within Bangladeshi aquaculture (oxytetracycline, chlortetracycline, amoxicillin trihydrate, sulfadiazine, sulfamethoxazole, trimethoprim, and doxycycline).

Permitting a limited number of AMs for use in aquaculture seems to be the better approach, as it is easier to regulate and track the use of a few compounds. However, such restrictions are easier to implement in countries that only produce a handful of species and with a strong consolidated industry. Further, this type of regulation does not necessarily restrict overuse of those AMs that remain allowed. Historically, the approved and banned AM lists available in low- and lower middle income countries have been based on food safety hazards and national or international export quality standards, while most high-income nations also consider potential risks to the environment and their efficacy to kill target fish pathogens as key criteria for their acceptance and registration of AMs.

The inconsistent and poor quality of the AM products available to the aquaculture industry in certain regions (e.g. Phu et al. 2015) presents a serious concern. In these regions, AMR could occur because the product label does not accurately describe the product. The global extent of this problem is unknown, but lack of quality control could have serious implication on animal and human health.

### Vaccines

Vaccines can efficiently prevent bacterial disease outbreaks in finfish, but they do not work in the same way for crustaceans or mollusks, as they do not have an adaptive immune system (Du Pasquier 2001). A prime example of the success of vaccines is salmon farming in Norway (Fig. 2), which over the past four decades managed to develop effective vaccines for most important bacterial diseases. While infectious diseases still cause mortality in the region, these are primarily caused by viruses [e.g. Pancreas Disease (PD), Heart and Skeletal Muscle Inflammation (HSMI), and Cardiomyopathy Syndrome (CMS)], against which AMs are not effective (Bondad-Reantaso et al. 2012). Vaccines have also been successfully implemented to treat Grass Carp Hemorrhagic Virus (GCHV) in China, where AM use is widespread throughout the Southern parts of the country (Mi et al. 2013). In other aquaculture sectors, vaccines have been less successful, as the cost of development and administration remains high (Secombes 2008). For example, a number of vaccines have been designed and commercialized against *Piscirickettsia salmonis* in Chile with low to moderate efficacy (Marshall and Tobar 2014), and Vietnamese farmers have shown overall skepticism to pangasius vaccines due to high costs, extensive labor efforts to inject individual fish, and limited survival improvements (Phu et al. 2016).

In conclusion, the success of vaccines in Norway was due to predominantly bacterial pathogens, high vaccine efficacy, and sufficient resources. For GCHV in China, vaccines have also been a success, with a cost–benefit ratio of 1:7 (Mi et al. 2013). While GCHV is a viral pathogen, reduced symptoms of disease and mortality rates will surely reduce the number of misdiagnoses and consequent AM use. The situations in Chile and Vietnam remain more difficult, suggesting that vaccines will not be a ‘silver bullet’ for all pathogens. The approaches towards designing new vaccines are, however, constantly developing, with the potential for considerably cheaper vaccines with higher efficacy in the future (Secombes 2008).

### Regulations

Regulations are typically applied to either direct use of AMs or the level of AMs in products. Direct use regulations include mandates on how specific AMs can be applied and under which circumstances. Regulation of AM use in northern European salmon farms has subsequently contributed to lower usage relative to other countries with less stringent regulations (Burridge et al. 2010).

In our opinion, product-based food safety regulations have been among the most effective drivers for reducing AM use in aquaculture to date. Product-based regulations are generally applied in the form of maximum residue limits, where samples of seafood products are screened for detectable levels of banned compounds or high concentrations of regulated compounds (Costello et al. 2001). In addition to regulations governing access to AMs (as discussed in Sect. “Limiting AM access”), national level regulations can also limit the amount of allowable AM residues in products meant for human consumption, thereby reducing overuse and misuse (Bondad-Reantaso et al. 2012). Screening consignments for AM residues is, however, resource intensive, meaning only a small sample of all products are often tested.

Over the past two decades, more stringent AM regulations can be associated with reduced use in European and North American aquaculture (given that Atlantic salmon makes up about half of production in these regions; FAO 2016a; Fig. 2) and possibly also reduced use in aquaculture production providing imports to these regions (Rico et al. 2013; Henriksson et al. 2015). However, import regulations do not address AM use throughout the production cycle, as farmers can limit AM concentrations in products by shifting the time of application or active substance. Instead, it would be more comprehensive to require farmers to register the quantities of AMs applied throughout the grow-out period, as is the case in Norway, Scotland, Chile, and some Canadian provinces (Burridge et al. 2010). This, however, requires a certain level of regulatory capacity to keep track of AM sales and use, and enforce accurate record keeping.

Product regulations have been shown to be effective at reducing at least late stage use of AMs in internationally traded products. For example, in response to regulation and monitoring of export-oriented products, contamination of Thai shrimp samples aimed for export dropped from 24% to 5% over a four-year period (Holmström et al. 2003). Product-based regulations can, however, incentivize different AM use practices among farmers dedicated to domestic or less regulated international markets. Similarly, differences in food safety standards between import countries (e.g. US vs. EU) can influence AM use practices in producing countries. Consequences of AM residues in seafood products also differ between importing countries, from consignments being held and thereby often lost, to bans on all animal imports from the country of origin (McCracken et al. 2013). In some cases, repeatedly stopped consignments have forced some countries to modify national regulations and AM use practices. For example, enrofloxacin has been banned in Vietnam since 2012 due to the repeated consignment rejections of pangasius catfish in the US (VMARD 2012), which has a zero tolerance limit.

Avoidance of food safety alerts have also resulted in better cooperation among aquaculture farmers, as processing plants and/or government officials now often screen consignments before shipping (caa.gov.in, accessed 02-May-2017; and pers. comm. processing plant managers in China and Indonesia). Thus, it is in all country-level producer’s interest to comply with regulations to enable product exports, especially to the EU and the US. However, these screenings and occasional consignments stopped by customs can become costly, rendering Europe or America less attractive as trading partners until regulations are enforced (Love et al. 2011; CBI 2013). For example, market prices for shrimp have been increasing in China with a doubling in consumption between 2005 and 2015,^1^ incentivizing many processing plants to shift their target market from the EU to China (CBI 2013). This could potentially result in less restrictive use of AMs, a shift that might be further influenced by the US withdrawal from the Trans Pacific Partnership.

Rapid unregulated expansions of aquaculture have also paved the way for disease outbreaks, with subsequent AM abuse as a consequence. The Chilean salmon industry, for example, grew rapidly for nearly 30 years before being hit by an outbreak of Infectious Salmon Anemia (ISA) (Bustos-Gallardo 2013). This outbreak was the consequence of a pursuit of increases in production, with the government failing to take into account scientific advice on disease risks (Bustos-Gallardo 2013).

### Certification

To comply with most types of certification, farmers need to limit and report on AM use. Existing organic certification standards have similar restrictions and all forbid prophylactic usage of AMs, but with some individual differences related to accepted practices and types of AMs (Table 1). The support provided on disease diagnostic and aquatic health management by certifying schemes can also influence the number and dosages used by farmers, as well as a number of issues related to record keeping, withdrawal periods, and food safety aspects.

**Table 1:** Standard rules proposed by the different certification schemes regarding AM use and management

							
Regulations	×	×	×	×	×	×	×
On-farm documentation	×	×	×	×	×	×	×
100% Veterinary prescription	×	×	×	×	×	×	×
No prophylactic	×	×	×	×	×	×	×
Reg. repeated treatments	–	–	×	–	×	×	×
Banned antibiotics	–	–	×	×	–	–	–
No human antibiotics	–	–	×	×	–	–	–
Env. monitoring	–	–	–	×	–	–	–

Banned antibiotics and some other rules might differ by country

*Sources* GLOBALGAP (globalgap.org); Safe Quality Food Institute (sqfi.com and fmi.org); GAA/ACC (gaalliance.org and aquaculturecertification.org); Naturland (naturland.de); DEBIO (debio.no); and KRAV (krav.se) (all accessed November 2016)

Market driven aquaculture certification is becoming an increasingly powerful tool to enforce compliance of the industry to international standards (Jonell et al. 2013). Market access and pricing strategies are slowly attracting more and more aquaculture farmers to adopt and implement Better Management Practices (BMPs) and Aquaculture Improvement Projects (AIPs) in order to comply with national and international standards, and transition to third party certification (e.g. ASC and BAP) (Jonell et al. 2013). BMPs also often promote farm level biosecurity and sustainable intensification practices, reducing the overall risks for disease (e.g. bapcertification.org).

News media can also help change AM use and improve production practices by influencing public perception, consumer awareness, and aquaculture firms’ ‘social license to operate’ (Leith et al. 2014). For example, several articles in popular press outlets have highlighted excessive use of AM drugs in Chilean salmon farming and Vietnamese pangasius farming.^2^ The coverage resulted in reduced purchases of Chilean salmon by consumers and decreases in the price of pangasius (Little et al. 2012). In this sense, the popular press can act as an enforcement mechanism for judicious use of AMs and general sustainability (Hosono et al. 2016), although it may also contribute to severe miscommunications (Murk et al. 2016).

Improved general public awareness would not only empower certification labels, but it would also help reduce unnecessary AM use among humans. Since human run-off is intermixed with aquaculture irrigation in many parts of the world, enlightening the public about responsible AM use would benefit people, animals, and the environment (Robinson et al. 2016).

## Outlook

Relative to the livestock sector, aquaculture is still highly diverse, thus complicating the application of top-down use estimates for quantifying species-based AM use. The expanding diversity of farmed aquaculture species also translates into a greater number of possible pathogens, especially for new species or rearing of established species in new environments. This can, due to the lack of historical perspectives, prevent farmers from correctly identifying and preventing disease. In these cases, AM usage often follows as the first line of defence. Species diversity, on the contrary, also adds resilience towards disease outbreaks, as it limits the impacts of emerging pathogens. Thus, maintaining species diversity in aquaculture could add resilience towards disease outbreaks and ultimately limit AM use (Troell et al. 2014), with reservation for species susceptible to disease (see Sect. “Species vulnerability”).

**Fig. 4:**
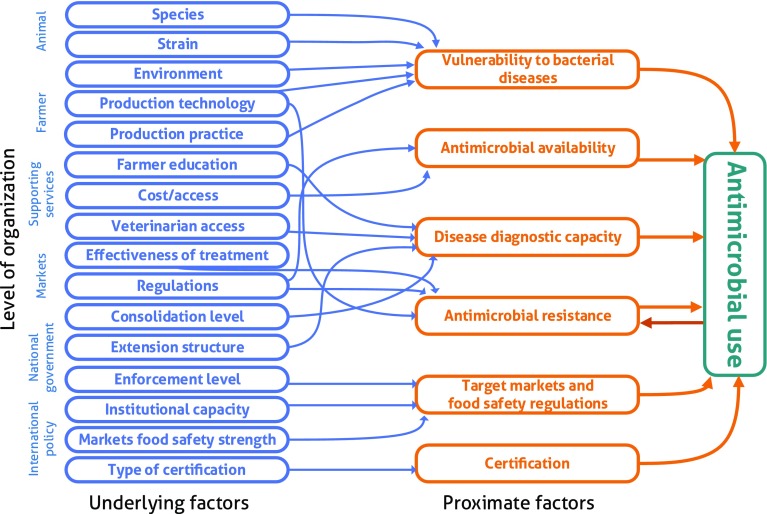
The figure visualizes the inter-linkages between underlying factors and AM use through a set of proximate factors. The underlying factors are organized from site-specific factors (e.g. animal and farmer), to those imposed at the international level. As each farm will be subject to a unique set of underlying factors, different combinations of proximate factors will up- or down-regulate consequent AM use. AM use itself also act on AMR, which could trigger additional AM use

In response to the fragmented global AM use data, we in Fig. 4 summarize the most influential underlying and proximate factors driving AM use in the aquaculture sector today. These factors range from the individual animal to the international policy level, with different levels of organization applicable to different seafood commodities. Excessive and repeated use of AMs in aquaculture also contribute to increased levels of AMR in the surroundings of the production facilities. The transmission of AMR genes by horizontal and vertical gene transfer, in turn, contributes to the selection of bacterial phenotypes with enhanced virulence and pathogenicity, which easily results in additional AM use.

Another difference between AM use in terrestrial livestock and aquaculture lies in their impact pathways on humans. Many terrestrial livestock are warm-blooded mammals that share much of our bacterial community, and thus many of our pathogens. Fish are cold-blooded and, for the most part, maintain different microbiota. A few fish bacterial pathogens such as *Mycobacterium* spp., *Streptococcus iniae*, *Clostridium botulinum*, and *Vibrio vulnificus* are known to be zoonotic; having the unique ability to cross phyla/class barriers to infect humans (Lehane and Rawlin 2000; Miller and Neely 2005; Gauthier 2015). AMR in these types of pathogens could have serious implications for treatment in infected individuals that have been in direct contact with the animals. The greater risk, however, may be associated with non-pathogenic bacteria in the environment. Several studies have shown that release of antibiotics from aquaculture facilities can increase the risk of AMR in environmental compartments (Tendencia and De La Peña 2001; Le et al. 2005; Sun et al. 2016; Nakayama et al. 2017; Rico et al. 2017) and that the aquatic environment is a source of AMR genes able to colonize human bacteria, which could have devastating effect at a population scale (Rhodes et al. 2000; Poirel et al. 2012; Aedo et al. 2014; García-Aljaro et al. 2014; Xu et al. 2017).

AMR gene transmission also goes beyond aquaculture production limits and become a universal issue. The problem of AM use and AMR therefore needs to be tackled internationally across all sectors by adopting one common broad-scale approach, which includes a coordinated ‘One Health’ perspective, where animal husbandry and human medicine practitioners are seen as inherently interconnected (AVMA 2008; onehealthinitiative.com, accessed 29-Aug-2017). In this regard, the pathways and sources of AMR gene flows among compartments and across productive systems need to be further investigated and better mapped (Wernli et al. 2017).

## Conclusions

While AM use seems to have considerably decreased in some farming sectors (e.g. Norwegian salmon and Thai shrimp), overall aquaculture production is most likely increasing and some production systems/areas that dominate global trade remain problematic (e.g. salmon in Chile, pangasius in Vietnam, and shrimp in India). However, apart from salmon farming, only fragmented quantitative data exist on AM use in the aquaculture industry. With regards to China, the world’s largest aquaculture producer, only a few production systems and provinces have available up-to-date AM records (Rico et al. 2013; Liu et al. 2017), and, to our knowledge, no noteworthy dataset exists on carp farming, the most commonly farmed finfish worldwide. Thus, it is impossible to accurately quantify AM use in aquaculture globally. Information on the quality of AMs used in aquaculture is also only available for certain regions, with no rigorous global estimates.

The lack of regulations in many low and lower middle income countries, or inadequate enforcement of existing regulations, has incentivized restrictions on AM residues in seafood imported to high income countries. Even though this is an important mechanism to limit AM use, it only applies to internationally traded products, and while seafood remains the most traded animal product, it leaves production aimed for domestic consumption largely unregulated. Moreover, changes in trade patterns due to political and dietary shifts might reduce current levels of controls if importing countries have weaker regulations.

Conclusively, field surveys and improved record keeping of AM sales on country and species bases are needed for establishing comprehensive AM use databases for aquaculture. Such databases should be used to identify global hotspots in which AMs are disproportionally used and that require urgent attention and better management. In addition, risk assessment approaches for preventing diseases, and the development and spread of AMR bacteria in aquatic environments need to be established. Identifying the two-way link between AM use in aquaculture and AMR in humans is also of critical importance as the aquatic environment often constitute the final receiver of both anthropogenic and livestock waste.

## Electronic supplementary material

Below is the link to the electronic supplementary material.

Supplementary material 1 (DOCX 42 kb)

## Footnotes

1
http://www.seafoodsource.com/commentary/china-is-poised-to-dictate-asian-seafood-supply. Accessed: 23-Oct-2016.

2 For example: nytimes.com/2009/07/27/world/americas/27salmon.html and http://www.reuters.com/article/us-usa-catfish-idUSKBN0TF0BN20151126.
