# Supplementary material for: Unpacking factors influencing antimicrobial use in global aquaculture and their implication for management: a review from a systems perspective
Source: Sustain Sci. 2017 Nov 18;13(4):1105–20. doi: 10.1007/s11625-017-0511-8 (PMC6086308; doi:10.1007/s11625-017-0511-8)
Supplement: Supplementary file 1 — Supplementary material 1 (DOCX 42 kb) [file 11625_2017_511_MOESM1_ESM.docx]

**Supporting Material** – **Unpacking factors influencing antimicrobial use in global aquaculture and their implication for management**

Patrik JG Henriksson, Andreu Rico, Max Troell, Dane Klinger, Alejandro H Buschmann, Sonja Saksida, Mohan V Chadag and Wenbo Zhang

Table S1: Banned therapeutants by China’s government (Ministry of Agriculture). Source：(Zhang 2014)

| Name (CN) | Name(EN) |
| --- | --- |
| 地虫硫磷 | Fonofos |
| 六六六 | Benzem,Bexachoridge |
| 林丹 | Lindane,Agammaxare, Gamma-Bhc Gamma-Hch |
| 毒杀芬 | Camphechlor(Iso) |
| 滴滴涕 | Ddt |
| 甘汞 | Calomel |
| 硝酸亚汞 | Mercurous Nitrate |
| 醋酸汞 | Mercuric Acetate |
| 呋喃丹 | Carbofuran |
| 杀虫脒 | Chlordimeform |
| 双甲脒 | Anitraz |
| 氟氯氰菊酯 | Flucythrinate |
| 五氯酚钠 | Pcp-Na |
| 孔雀石绿 | Malachite Green |
| 锥虫胂胺 | Tryparsamide |
| 酒石酸锑钾 | Anitmonyl Potassium Tartrate |
| 磺胺噻唑 | Sulfathiazolum St,Norsultazo |
| 磺胺脒 | Sulfaguanidine |
| 呋喃西林 | Furacillinum, Nitrofurazone |
| 呋喃唑酮 | Furazolidonum, Nifulidone |
| 呋喃那斯 | Furanace, Nifurpirinol |
| 氯霉素(包括其盐、酯及制剂) | Chloramphennicol |
| 红霉素 | Erythromycin |
| 杆菌肽锌 | Zinc Bacitracin Premin |
| 泰乐菌素 | Tylosin |
| 环丙沙星 | Ciprofloxacin (Cipro) |
| 阿伏帕星 | Avoparcin |
| 喹乙醇 | Olaquindox |
| 速达肥 | Fenbendazole |
| 乙烯雌酚 | Diethylstilbestrol, Stilbestrol |
| 甲基睾丸酮 | Methyltestosteronum |

Table S2: Permitted antibiotics by China’s government (Ministry of Agriculture). Source: Chen & Wang, 2015.

| Category(CN) | Name(CN) | Category(EN) | Name(EN) |
| --- | --- | --- | --- |
| 氨基糖苷类 | 硫酸新霉素（粉） | Amino glycosides | Neomycin sulfate (powder) |
| 四环素类 | 盐酸多西环素（粉） | Tetracycline | Doxycycline hydrochloride (powder) |
| 酰胺醇类 | 甲砜霉素（粉） | Amphenicols | Thiamphenicol (powder) |
|  | 氟苯尼考（预混剂） |  | Florfenicol (premix) |
|  | 氟苯尼考（粉） |  | Florfenicol (powder) |
| 磺胺类药物 | 复方磺胺嘧啶（粉） | Sulfa drugs | Compound sulfadiazine (powder) |
|  | 复方磺胺甲恶唑（粉） |  | Compound sulfamethoxazole (powder) |
|  | 复方磺胺二甲嘧啶（粉） |  | Compound sulfamethazine (powder) |
|  | 磺胺间甲氧嘧啶钠（粉） |  | Sulfamonomethoxine sodium (powder) |
|  | 复方磺胺嘧啶（混悬液） |  | Compound sulfadiazine (suspension) |
| 喹诺酮类药物 | 恩诺沙星（粉） | Quinolones | Enrofloxacin (powder) |
|  | 乳酸诺氟沙星（粉） |  | Lactate norfloxacin (powder) |
|  | 诺氟沙星（粉） |  | Norfloxacin (powder) |
|  | 烟酸诺氟沙星（预混剂） |  | Niacin norfloxacin (premixes) |
|  | 诺氟沙星盐酸小檗碱（预混剂） |  | Norfloxacin berberine hydrochloride (premixes) |
| 噁喹酸 | 噁喹酸（散） | oxolinic acid | Oxolinic acid (powder) |
|  | 噁喹酸（混悬液） |  | Oxolinic acid (suspension) |
|  | 噁喹酸（溶液） |  | Oxolinic acid (solution) |
|  | 氟甲喹（粉） |  | Flumequine (powder) |
|  | 盐酸环丙沙星盐酸小檗碱 （预混剂） |  | Ciprofloxacin hydrochloride berberine (premixes) |
|  | 维生素c磷酸酯镁盐酸环丙沙星（预混剂） |  | Vitamin c, magnesium phosphate Ciprofloxacin (premixes) |

Table S3: Antibiotics found in China’s aquaculture environment

Source: (Huang et al. 2016)

| Year | Antibiotics | Province | Sample |
| --- | --- | --- | --- |
| 2013 | Norfloxacin, ofloxacin, enrofloxacin, tetracycline, erythromycin dehydration | Guangdong | Freshwater aquaculture Sediment |
| 2009 | Norfloxacin, ciprofloxacin, enrofloxacin | Guangdong | Freshwater aquaculture Sediment |
| 2009 | Norfloxacin, ciprofloxacin | Guangdong | Brackish water aquaculture Sediment |
| 2011 | Sulfamethoxazole, sulfamethoxazole thiadiazole, sulfadimethoxine, norfloxacin | Tianjin | Freshwater aquaculture Sediment |
| 2014 | Sulfadiazine, sulfamethazine, sulfamethoxazole | Guangdong | Brackish water aquaculture Sediment |
| 2013 | Norfloxacin, ofloxacin, tetracycline | Guangdong | Freshwater aquaculture Sediment |
| 2011 | Ciprofloxacin, enrofloxacin, and oxytetracycline | Tianjin | Freshwater aquaculture Sediment |
| 2011 | Norfloxacin, sulfamethazine, sulfamethoxazole, Florfenicol, ofloxacin | Fujian | Brackish water aquaculture Sediment |
| 2011 | Norfloxacin, ofloxacin, sulfadiazine, sulfamethazine, sulfamethoxazole, Florfenicol, trimethoprim | Fujian | Brackish water aquaculture Sediment |

| Table S4: Reported incidents involving AMs by country from the EU’s Rapid Alert System for Food and Feed, the US’s Food and Drug Administration, and Japan’s Ministry of Health, Labour and Welfare, between 2000 and 2015 | | | | | | | | | | | | | | | | |
| --- | --- | --- | --- | --- | --- | --- | --- | --- | --- | --- | --- | --- | --- | --- | --- | --- |
|  | 2000 | 2001 | 2002 | 2003 | 2004 | 2005 | 2006 | 2007 | 2008 | 2009 | 2010 | 2011 | 2012 | 2013 | 2014 | 2015 |
| Austria |  |  |  |  |  |  |  |  |  |  |  | 1 |  |  |  |  |
| Belgium |  |  | 2 |  |  |  |  |  |  |  | 5 | 1 |  |  |  | 1 |
| Brazil |  |  |  |  |  |  |  |  |  |  |  |  | 1 |  |  |  |
| Canada |  |  |  |  |  | 1 |  |  |  |  |  |  |  |  |  |  |
| Chile |  |  |  |  | 1 |  |  |  |  |  |  | 1 |  |  |  |  |
| China |  | 37 | 33 |  | 3 | 8 | 45 | 58 | 14 | 20 | 26 | 12 | 6 | 3 | 5 | 1 |
| Czech Republic | |  |  |  |  |  |  |  |  | 1 |  |  |  | 1 |  |  |
| Denmark |  |  |  |  | 3 | 2 |  |  |  |  |  | 1 |  |  |  |  |
| France |  |  | 1 |  |  |  |  |  |  |  | 6 |  |  |  |  | 1 |
| Germany |  |  |  |  | 1 |  |  |  |  |  |  |  | 1 |  |  |  |
| Greece |  |  |  |  |  | 2 | 1 | 1 |  |  |  |  |  |  |  | 3 |
| Hong Kong |  |  |  |  |  |  |  |  |  |  | 1 |  |  |  |  |  |
| India |  |  | 3 |  |  |  | 3 |  |  |  |  | 3 | 7 | 5 | 12 | 7 |
| Indonesia | 1 | 5 | 4 |  | 1 | 5 | 26 | 36 | 11 | 2 |  |  | 2 |  |  |  |
| Italy |  |  | 1 |  |  |  |  |  |  | 1 |  |  |  |  |  |  |
| Jamaica |  |  |  |  |  |  |  | 1 |  |  |  |  |  |  |  |  |
| Japan |  |  |  |  | 2 |  |  |  |  |  |  |  |  |  |  |  |
| South Korea | |  | 1 |  | 1 |  |  | 9 | 10 | 4 | 2 | 4 | 4 | 2 |  |  |
| Latvia |  |  | 1 |  |  |  |  |  |  |  |  |  |  |  |  |  |
| Malaysia |  |  |  |  |  | 1 |  |  |  |  |  |  |  |  |  |  |
| Myanmar |  |  | 1 |  |  |  | 1 |  |  |  |  |  |  |  |  |  |
| Netherlands |  |  |  |  |  | 3 | 2 |  |  | 1 | 2 |  |  |  | 1 |  |
| Pakistan |  |  | 2 |  |  |  |  |  |  |  |  |  |  |  |  |  |
| Peru |  |  |  |  | 14 | 3 | 1 |  |  |  |  |  |  |  |  |  |
| Poland |  |  |  |  |  |  |  |  |  | 1 | 1 | 1 |  | 1 |  |  |
| Slovakia |  |  |  |  |  |  |  |  |  |  |  |  | 1 |  |  |  |
| Spain |  |  |  |  |  |  | 1 |  |  |  |  |  |  |  |  |  |
| Taiwan |  |  |  | 1 | 1 |  | 6 | 9 | 3 | 4 | 4 | 1 |  |  |  |  |
| Thailand |  |  | 19 |  |  |  | 1 | 4 | 2 |  | 2 | 2 |  | 1 |  |  |
| Turkey |  |  |  |  |  |  |  |  |  | 1 |  |  | 1 |  |  |  |
| Unknown origin | | 7 | 3 |  |  |  |  |  |  |  |  |  |  |  |  |  |
| Vietnam |  | 27 | 19 | 1 | 12 | 51 | 86 | 124 | 46 | 33 | 36 | 104 | 55 | 35 | 47 | 32 |

**References**

Chen, C., & Wang, Y. (2015). Aquaculture antibiotics use Situation, Problems and Countermeasures (Part one) (in Chinese). *China Fisheries*, (4), 65–68.

Huang K, Zhao D, Yang H, et al (2016) Research progress on pharmaceuticals and personal care products (PPCPs) of fishery environment and aquatic products (in Chinese). South China Fish Sci 12:119–126.

Zhang W (2014) SUSTAINING EXPORT-ORIENTED VALUE CHAINS OF FARMED SEAFOOD IN CHINA. University of Stirling
